# Recurrence of Cholesteatoma Mimicking Dural Herniation

**Published:** 2011

**Authors:** Mohsen Rajati Haghi, Seyyed Masood Naseri Sadr

**Affiliations:** 1 *Ear, Nose, Throat, Head and Neck Surgery Research Center, Department of otorhinolaryngology, Mashhad University of Medical Sciences, Mashhad, Iran*; 2 *Department of otorhinolaryngology, Mashhad University of Medical Sciences, Mashhad, Iran*

## Case Report

A 25 year old man was admitted to otorhinolaryngology department with a mass in the ear canal. The patient had a history of tympanoplasty with mastoidectomy in another centre (for chronic otorrhea)

2 years earlier. Details of the surgery were not available. He had no discharge during this time and he came to our hospital for routine follow-up visits. On otomicroscopy a white mass was noticed in the superior part of the canal, the origin of which could not exactly be localized. On palpation with a blunt instrument it proved to be soft. No otorrhea was noted in the canal and the tympanic membrane seemed to be intact in the visible area.

The most likely diagnosis was cholesteatoma recurrence but the chief differential diagnosis to be ruled out in such cases is meningocele and dural herniation.

An HRCT was ordered and the mass origin from attic was obvious. The intact tegmen tympani rules out intracranial involvement. The patient was operated and a rather extensive cholesteatoma was found and cleaned. It seems that he has had an attic cholesteatoma which went unnoticed and consequently unmanaged in the previous surgery and tympanoplasty had been done on the epidermal remnants in the attic.

**Fig 1:**
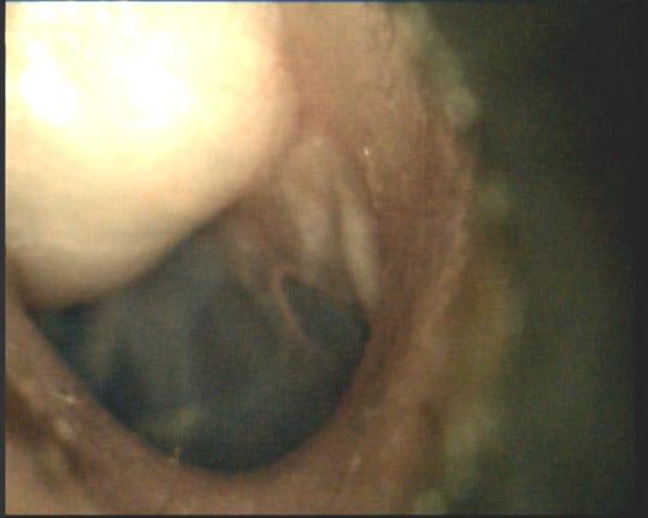
Otoscopic view

**Fig 2:**
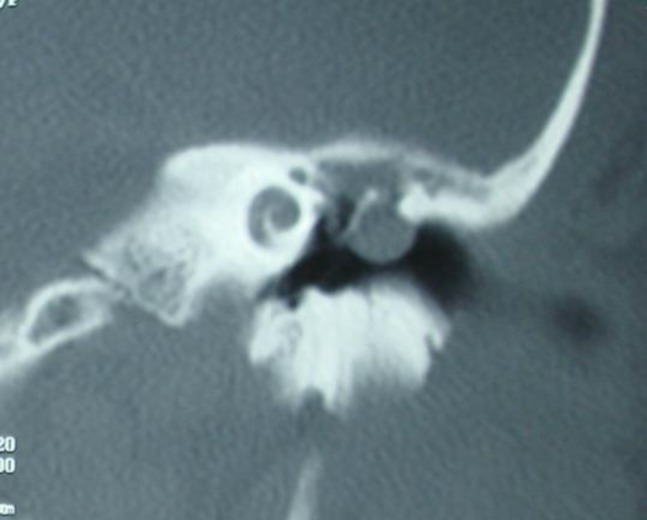
Coronal HRCT

